# Statistical Optimization of Hydrothermal Conversion of *Stevia rebaudiana* Residues for Sustainable Production of 5-HMF and Furfural as Platform Chemicals

**DOI:** 10.3390/plants15050830

**Published:** 2026-03-08

**Authors:** Koray Alper, Sinem Çolak

**Affiliations:** Department of Chemical and Chemical Processing Technologies, Çaycuma Food and Agriculture Vocational School, Zonguldak Bülent Ecevit University, Zonguldak 67600, Türkiye; sinem.colak@beun.edu.tr

**Keywords:** hydrothermal liquefaction, *Stevia rebaudiana*, 5-hydroxymethylfurfural, furfural, SEM, FT-IR, statistical optimization

## Abstract

In recent years, the sustainable production of bio-based platform chemicals from non-lignocellulosic biomass has garnered increasing attention. In this study, *Stevia rebaudiana* residues were evaluated via hydrothermal liquefaction (HTL) to produce key furan derivatives, namely 5-hydroxymethylfurfural (5-HMF) and furfural. The effects of reaction temperature (160–240 °C) and pressure (0–8 MPa) on product yields were systematically investigated and statistically evaluated using Analysis of Variance (ANOVA) and regression modeling. The highest 5-HMF (93.1 mg/L) and furfural (51.2 mg/L) yields were obtained at 200 °C, while pressure was found to have no statistically significant effect on product formation. To elucidate the physicochemical transformations occurring during hydrothermal processing, Scanning Electron Microscopy (SEM) and Fourier Transform Infrared (FT-IR) spectroscopy were used to analyze the morphological and functional group evolution of the biochar and bio-oil fractions. SEM images revealed gradual structural degradation, pore formation, and carbonization with increasing temperature, while FT-IR analysis confirmed dehydration, hydrolysis of glycosidic bonds, aromatization, and the formation of carbonyl groups directly related to furan production. A validated High-Performance Liquid Chromatography (HPLC-UV) method providing analytical efficiency for the precise determination of 5-HMF and furfural in complex biomass matrices was developed. This study provides a comprehensive understanding of the thermochemical behavior of *Stevia rebaudiana* biomass by integrating morphological characterization, molecular-level spectroscopy, and statistical process modeling and establishes a predictive framework for optimizing furan production under hydrothermal conditions. The findings highlight the potential of *Stevia rebaudiana* residues as a sustainable feedstock within circular bioeconomy strategies and offer a scalable approach for converting agricultural waste into high-value platform chemicals.

## 1. Introduction

*Stevia rebaudiana*, a perennial herbaceous plant belonging to the Asteraceae family and originally native to Paraguay in South America, has gained global importance as a low-calorie natural sweetener, with widespread use across Asia and several countries of the European Union; currently, Japan and Korea represent major consumer markets, while China stands as the leading global producer, accounting for nearly 80% of total stevia exports [1,2]. *Stevia rebaudiana* has been widely used for decades as a natural sweetener in the food and beverage industry because its leaves contain stevioside and rebaudioside and its cultivation has expanded in recent years due to its increasing use in the food industry [3]. *Stevia rebaudiana*, a natural non-caloric sweetener, is widely used as a substitute for sugar and belongs to a genus comprising about 150 species of herbs and shrubs [4]. Steviol glycosides, natural-origin non-caloric sweeteners, can serve as a versatile alternative to artificially synthesized sweeteners, helping to lower the risk of diabetes, caries, obesity, cardiovascular disease, and other diet-related illnesses [5]. Stevioside and rebaudioside A are 300–400 times sweeter than sucrose, calorie-free, and safe for individuals with type II diabetes, phenylketonuria, or Candida infections [6]. Fructans, another commercially significant metabolite, have been extracted from the roots of *Stevia rebaudiana* [7]. *Stevia rebaudiana* biomass is a renewable resource with carbon-neutral properties, as it absorbs carbon dioxide during its growth phase, helping to offset carbon dioxide emissions [8]. There are many studies in literature that utilize the biomass of the *Stevia rebaudiana*. Kargar and colleagues investigated the use of *Stevia rebaudiana* as a potential green synthesis agent for colloidal nanoparticles due to its antioxidant, non-toxic, low-cost, and stabilizing properties. In their study, silver nanoparticle (Ag NP) colloids were synthesized using extracts from *Stevia rebaudiana* plant leaves. The synthesized Ag NPs were characterized and evaluated for their performance as an affordable, biological, and readily available catalyst. This catalyst was employed for the selective synthesis of 5-hydroxymethyl-2-furan carboxylic acid (HMFCA) from the platform molecule 5-hydroxymethylfurfural (5-HMF). The process yielded an impressive 98% HMFCA in just 45 min at 50 °C [9]. Ansari and Kahrizi synthesized carbon dots (C-dots), which are carbon-based nanomaterials typically smaller than 10 nm in diameter, using *Stevia rebaudiana*, a plant-based material, in a green, eco-friendly, and cost-effective manner. Carbon dots (C-dots) are utilized in various fields such as nanotechnology, biotechnology, optoelectronics, and environmental engineering. The hydrothermal carbonization of *Stevia rebaudiana* was carried out under mild conditions without using any chemical oxidizing agents. The morphological properties, surface groups, chemical composition, and structure of *Stevia rebaudiana*-based C-dots were analyzed using techniques such as SEM-EDS, TEM, fluorescence, FT-IR, UV-Vis, and XRD. These plant-based C-dots have been shown to be ideal, non-toxic, and promising markers for cellular biomolecular imaging [10]. Heo and colleagues provide significant insights into the cost-effective and sustainable production of 2,5-furandicarboxylic acid (FDCA) using raw plant materials rich in inulin. FDCA, derived from biological sources, can serve as a key material for plastic production. *Stevia rebaudiana*, with approximately 36% inulin content, is a promising plant-based source for FDCA production [11]. Rodríguez and colleagues synthesized γ-Al_2_O_3_ nanorods using an easy, low-cost, and surfactant-free synthesis method, with *S. rebaudiana* leaf extract employed to guide the formation of the porous structure. The mixture was dried at 80 °C and then calcined at 650 °C, resulting in alumina powders with a high surface area (357 m^2^/g) and uniform pore sizes. The diameter of the synthesized hollow nanorods ranged from 13 to 25 nm, while their length varied between 40 and 50 nm [12]. Yılmaz and colleagues synthesized silver nanoparticles (AgNPs) using an AgNO_3_ solution and *Stevia rebaudiana* leaf extract. The nanoparticles were determined to be spherical and polydisperse, with diameters ranging between 2 and 50 nm, and a maximum diameter of 15 nm. The ^1^H NMR spectrum of the silver nanoparticles synthesized with *Stevia rebaudiana* leaf extract revealed the presence of aliphatic CH_2_ groups, aliphatic and alcoholic CH_3_ groups, as well as aromatic components. The role of ketones in the formation of metal nanoparticles is discussed in the context of *Stevia rebaudiana* leaf extract, which lacks detectable enzymes or proteins [13].

While *Stevia rebaudiana* has been extensively researched for its sweetening compounds and green synthesis applications, its thermochemical valorization via hydrothermal liquefaction (HTL) remains largely unexplored. Specifically, systematic studies integrating statistical process modeling with detailed physicochemical characterization to evaluate furan production from Stevia-derived biomass are lacking. Previous research has primarily focused on product identification or catalyst-driven conversions, while the combined effect of temperature and pressure under controlled hydrothermal conditions has not been rigorously quantified. Furthermore, limited attention has been paid to establishing predictive statistical relationships between process variables and furan yields. Therefore, the current study aims to address this research gap by combining hydrothermal conversion of *Stevia rebaudiana* residues under systematically varied temperature and pressure conditions with ANOVA-based statistical modeling and morphological and spectroscopic analyses (SEM and FT-IR). By integrating process optimization with structural characterization, this study provides a comprehensive assessment of furan formation behavior and contributes to the sustainable valorization of Stevia biomass within circular bioeconomy strategies.

## 2. Experimental

### 2.1. Chemical and Materials

*Stevia rebaudiana* leaf powder (foodgrade) was supplied from a local market in Karabük, Turkey. High-purity 5-HMF (Merck, Darmstadt, Germany, 99%) and furfural (Acros-Organics, Geel, Belgium,99%) were preferred for use in creating the calibration. Ultrapure water, with a conductivity of 18.3 MΩ·cm^−1^, was produced using a Zeneer Power I system (Human Corporation, Seoul, Republic of Korea) and utilized in the preparation of the mobile phase. Additionally, HPLC-grade methanol (≥99.9%) was procured from Sigma-Aldrich (St. Louis, MO, USA) and employed as a component of the mobile phase. Solid phase extraction (SPE) was performed using a Sep-Pak C18 cartridge produced by Waters Corporation (Milford, MA, USA).

### 2.2. Experimental Procedure

Fuels and valuable compounds have been obtained from biomass using hydrothermal methods in a high-pressure reactor. The reactor features a 500 mL stainless steel chamber, capable of operating at temperatures up to 500 °C and pressures up to 34.5 MPa. It is equipped with a motorized stirrer with adjustable speed and an integrated cooling system. Temperature and pressure are monitored through an integrated thermocouple and barometer, respectively. To ensure an inert atmosphere during experiments, nitrogen gas is supplied to the system. In the hydrothermal liquefaction process, 10 g (on a dry basis) of *Stevia rebaudiana* powder was introduced into the reactor vessel, and 150 mL of ultra-pure water was added prior to sealing the system. To ensure an oxygen-free environment, nitrogen gas was purged into the sealed chamber. The experiments were conducted at temperatures of 160, 180, 200, 220, and 240 °C for a duration of 15 min under an initial gas pressure of 0 MPa. To optimize the yield of 5-HMF and furfural, additional experiments were carried out at varying pressures of 1, 2, 4, and 8 MPa.

All experiments were repeated three times, and the standard deviation values are indicated in the error bars of the product distributions. After the reactions were completed, the reactor was cooled to room temperature using a water-cooling unit. Before opening the reactor, the accumulated gas inside was released. The mixture of solid and liquid products in the reactor was transferred to a beaker. The solid and liquid products were separated using vacuum filtration with a Büchner funnel and medium-grade qualitative filter paper. The liquid portion was taken into a separation funnel and extracted with dichloromethane (150 mL). Anhydrous sodium sulfate (Na_2_SO_4_) was added to the obtained solution for drying. Then, the Na_2_SO_4_ was filtered out, and the DCM solution in a round-bottom flask was evaporated using a rotary evaporator at 40 °C. After the dichloromethane was eliminated, the amount of the remaining fraction was determined and labeled as bio-oil. After separating the liquid and solid products, the solids remaining on the filter paper were dried in an oven at 105 °C for 4 h until constant weight was achieved. The dried solids were then weighed and defined as biochar.

After the bio-oil was obtained through liquid–liquid extraction, the remaining liquid product was subjected to solid-phase extraction. HPLC analysis was performed to determine the amount of 5-HMF, a platform compound derived from biomass sources. The solid-phase extraction process was carried out using Sep-Pak C18 cartridges. The cartridge is conditioned by passing 2 mL each of methanol and water. After conditioning, the cartridge is loaded with 1 mL of the liquid product obtained from biomass. The cartridge is dried with dry air. The cartridge was eluted with 2 mL of methanol, transferred to the vial and analyzed by HPLC-UV after passing a 0.45 μm membrane. Solid-phase extraction (SPE) using Sep-Pak C18 cartridges was employed to purify the bio-oil samples prior to HPLC analysis. This step effectively removed interfering compounds (e.g., sugars, lignin derivatives) that could co-elute with 5-HMF and furfural.

### 2.3. HPLC Instrumentation and Chromatographic Conditions

**Figure 1:**
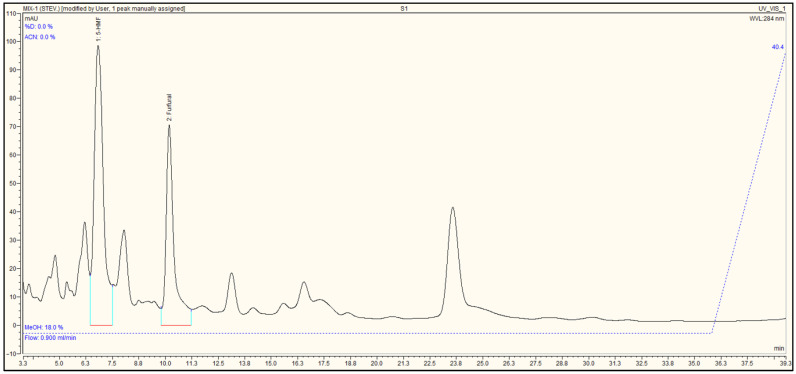
5-HMF and furfural chromatograms.

5-HMF analyses were performed using HPLC (Ultimate 3000, Thermo Dionex, Sunnyvale, CA, USA ) equipped with a UV detector, pump system, and an automatic sample injector. The analyses were carried out using a C18 column (Acclaim 120, 5 µm, 120 Å, 4.6 × 250 mm; Thermo Fisher Scientific, Sunnyvale, CA, USA). As described in the study by Ariffin et al., a methanol/water mixture was chosen as the mobile phase [14]. The injection volume for the instrument were set to 10 µL, the mobile phase was a methanol:water (18:82, *v*/*v*) mixture, and the flow rate was determined to be 0.9 mL per minute. 5-HMF and furfural was detected at a wavelength of 284 nm using the UV detector. Peak areas were determined using the chromatographic software of the HPLC system with automated baseline integration. Quantification was performed based on external calibration curves generated from analytical standards. The mobile phase composition was optimized to achieve baseline separation of 5-HMF (retention time: 7.173 min) and furfural (retention time: 10.053 min) within a 40 min run time. This ratio was selected based on prior studies indicating that methanol-water mixtures improve peak resolution for polar biomass-derived compounds [15,16]. The use of a C18 column (Thermo Acclaim 120, 5 µm, 120 Å) ensured high selectivity, while the low flow rate (0.9 mL/min) minimized backpressure and column degradation. Figure 1 presents the 5-HMF and furfural chromatograms. All sample solutions were filtered through 0.45 µm filters before being injected into the instrument. The data were processed using the licensed Chromeleon Client software (Dionex Corporation, USA). Our HPLC method achieves comparable LOD/LOQ values (Table 1 and Table 2) to prior studies [15], but with a shorter runtime (40 min vs. 60 min), enhancing throughput. Additionally, our method achieved a linear range for 5-HMF between 2.5–500 mg/L (Table 1), which is consistent with validated protocols for biomass hydrolysates [14,17]. The LOD and LOQ values are comparable to those reported for agricultural-industrial matrices and provide reliability in complex samples.

### 2.4. Calibration Curves, Limit of Detection and Quantification

Stock 5-HMF (5000 mg/L) and furfural (10,000 mg/L) solutions were prepared using methanol and stored in a refrigerator at 4 °C. Standard solutions were prepared by diluting the stock solutions, covering a concentration range of 2.5–500 mg/L. The correlation coefficient (r^2^), standard deviation (SD), linearity range, LOD and LOQ values for the calibrations prepared for 5-HMF and furfural are provided in Table 1 and Table 2. Based on the data presented in the tables, the method used for measuring 5-HMF and furfural is reliable.

### 2.5. Statistical Analysis of Experimental Data

The statistical evaluation of data obtained from experimental studies and the analysis of interactions between process parameters were performed using Minitab statistical software (Version 17, Minitab Inc., USA). ANOVA was applied to determine the individual effects of reaction temperature and pressure on 5-HMF and furfural yields and their statistical significance. The significance of the obtained models was tested at a 95% confidence interval (*p* < 0.05). Regression analysis was performed using the least squares method to mathematically describe the product formation associated with temperature increase and the subsequent observed thermal degradation behavior. In addition to linear models, quadratic polynomial models were also tested, and the model with the highest coefficient of determination (R^2^) was selected. The effect of the pressure parameter on the process was also verified using the same statistical methods.

## 3. Results and Discussion

### 3.1. Effect of Temperature and Pressure on Bio-Oil and Biochar Yields

**Figure 2:**
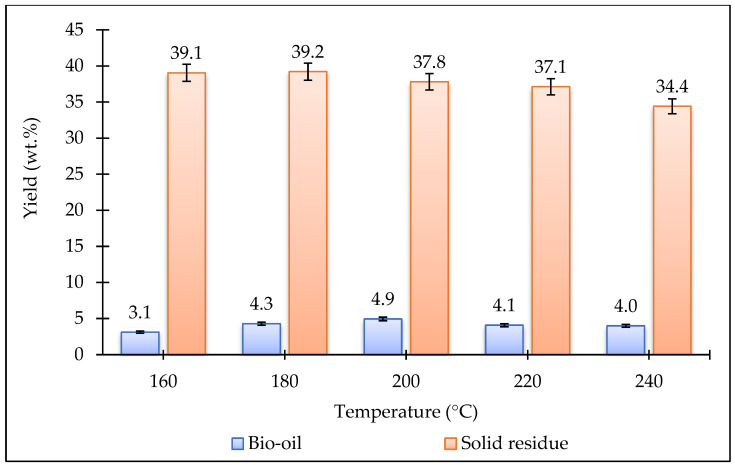
Bio-oil and biochar yields produced from HTL processing of *Stevia rebaudiana* at different temperatures (0 MPa, 15 min).

**Figure 3:**
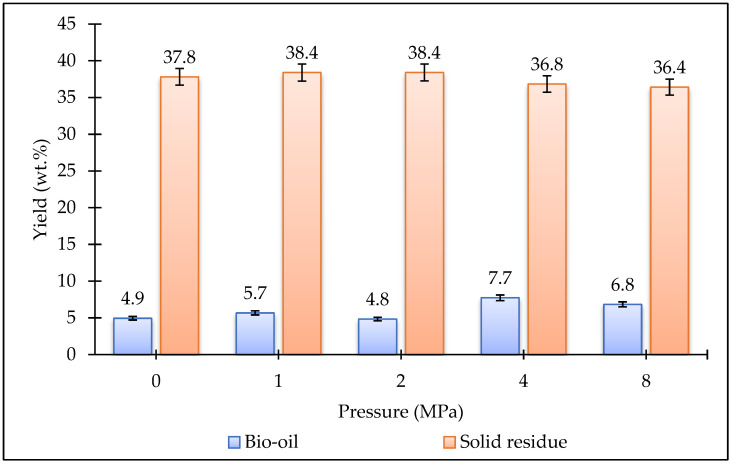
Bio-oil and biochar yields produced from HTL processing of *Stevia rebaudiana* at different pressures (200 °C, 15 min).

Figure 2 and Figure 3 show the effect of temperature and pressure on the bio-oil and biochar yields obtained from the HTL of the *Stevia rebaudiana* plant. As the temperature increased, the biochar yields gradually decreased. The lowest bio-oil yield (3.1 wt%) was obtained at 160 °C. When the temperature was increased from 160 °C to 240 °C, the bio-oil yield slightly increased, reaching 4.0 wt%. The highest bio-oil yield (4.9 wt%) was achieved at 200 °C. Experiments were conducted at different pressures at a constant temperature of 200 °C. Increasing the pressure did not have a significant effect on the biochar yields. However, bio-oil yields increased at higher pressures. The highest bio-oil yield (7.7 wt%) was obtained at 4 MPa pressure. Based on the data obtained in this study, the optimum temperature and pressure were determined to be 200 °C and 4 MPa, respectively. In future studies, catalyst experiments could be conducted under these optimum conditions to investigate changes in yields.

### 3.2. SEM Analysis of Biochar Morphology at Different Temperatures

**Figure 4:**
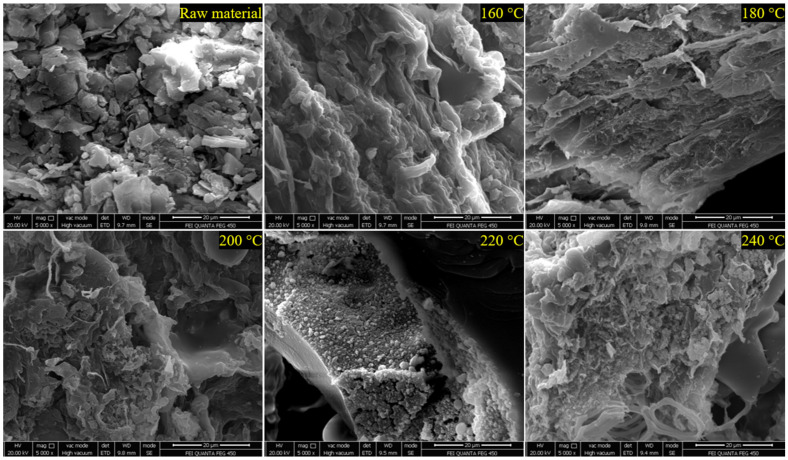
SEM image of *Stevia rebaudiana*-based HTC.

The thermally processed samples were analyzed using an FEI QUANTA FEG 450 SEM under high vacuum at an acceleration voltage of 20 kV and 5000× magnification. Images obtained in Secondary Electron (SE) mode were examined to reveal morphological changes on the surface. This study comprehensively investigated the morphological and structural changes in *Stevia rebaudiana* during thermal degradation using SEM analysis. Figure 4 clearly illustrates the progressive changes in biochar morphology as the treatment temperature increased from 160 °C to 240 °C. The findings demonstrate that the leaves maintained relatively stable structural integrity up to 160 °C, but showed significant degradation signs at temperatures above 180 °C. As temperatures exceeded 200 °C, crack formations and pore expansions became apparent, which could negatively affect the leaves’ water retention capacity and rehydration properties. At higher temperatures (220 °C and 240 °C), intense carbonization and amorphous structure formation were observed, likely resulting from the decomposition of organic components. This research provides valuable insights into the thermal behavior of *Stevia rebaudiana* and supplies essential data for optimizing its industrial applications.

### 3.3. FT-IR Analysis of Functional Groups in Bio-Oil and Biochar

**Figure 5:**
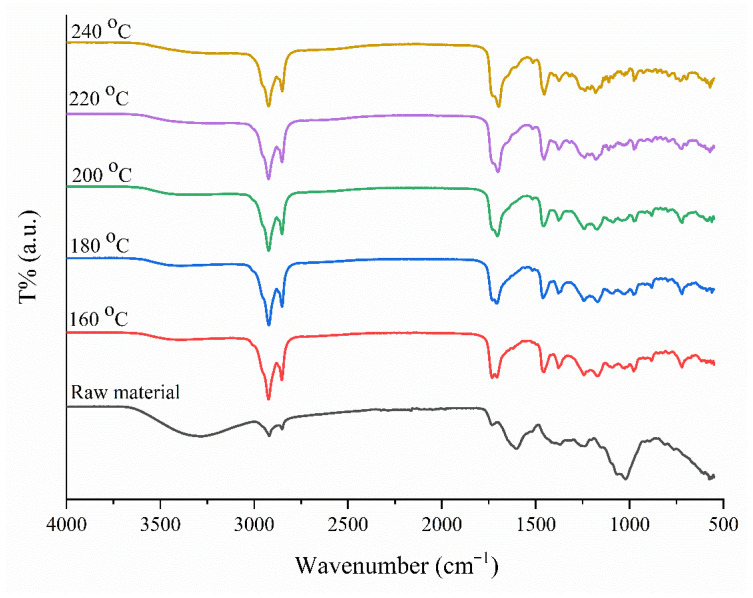
FT-IR spectra of bio-oil produced at different temperatures (160–240 °C) compared with the raw material.

The FT-IR spectra of the raw material and bio-oils obtained at different temperatures were examined (Figure 5), and it was observed that the complex structure of the biomass was broken down and new functional groups were formed during the hydrothermal process. A decrease was observed in the intensity of the broad O-H stretching vibrations in the 3200–3600 cm^−1^ range in the raw material spectrum. This indicates the removal of water through dehydration reactions occurring during the process [18]. The C-H stretching vibrations of the methyl (-CH_3_) and methylene (-CH_2_) groups are observed in the range of 2850–2950 cm^−1^ in bio-oil samples. The increase in the intensity of these peaks, which are quite weak in the raw material, proves that aliphatic carbon chains are formed in the structure along with the processing temperature and that the hydrocarbon content of the obtained product increases. The most critical finding of the analysis is the sharp C=O (carbonyl) peak that appears around 1700 cm^−1^ [19]. This peak, which is not observed in the raw material, represents the C=O (carbonyl) stretching vibrations in aldehydes, ketones, esters, and carboxylic acids. The presence of this peak spectrally confirms the formation of furan derivatives such as 5-HMF and furfural, which is the main objective of the study. Peaks around 1600 cm^−1^ indicate C=C vibrations in aromatic ring structures [20]. Additionally, in the fingerprint region between 1000–1300 cm^−1^, the replacement of broad bands in the raw material with sharper peaks indicates that the C–O–C glycosidic bonds present in the cellulose structure have been broken through hydrolysis [21]. When the temperature rises from 160 °C to 240 °C, the intensity of the aliphatic C-H (2900 cm^−1^) and carbonyl C=O (1700 cm^−1^) peaks is maintained. This indicates that at temperatures of 200 °C and above, biomass decomposes to a large extent, converting into liquid products with high chemical and energy density.

**Figure 6:**
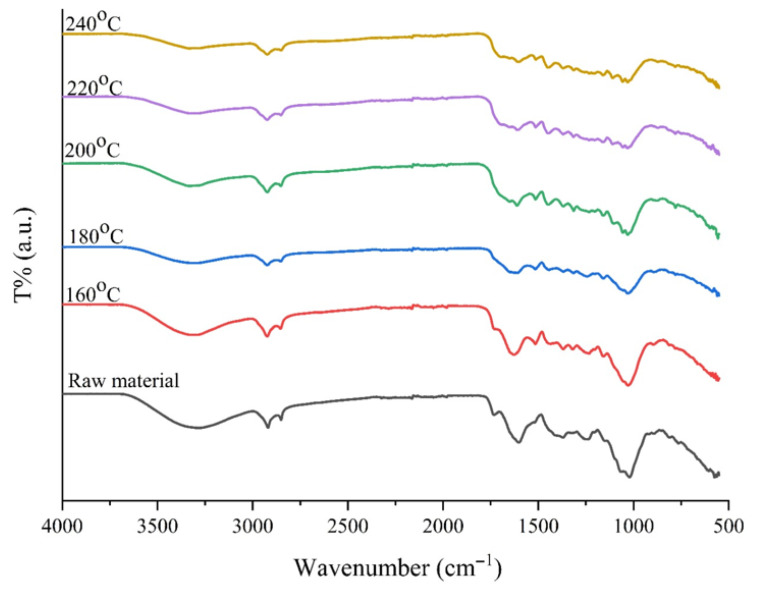
FT-IR spectra of biochar produced at different temperatures (160–240 °C) compared with the raw material.

The FT-IR spectra of raw biomass and biochar obtained at different temperatures (160–240 °C) are compared in Figure 6. Spectral changes demonstrate that the original structure of the biomass is disrupted and carbonization occurs as the intensity of the hydrothermal process increases. In the raw material, the wide and intense peak observed around 3400 cm^−1^ weakens significantly with increasing temperature, particularly at 220 °C and 240 °C; this change indicates that the structure reduces its oxygen content through dehydration (water loss). The most prominent change in the spectrum is the intense C-O stretching vibrations in the 1030–1050 cm^−1^ region, which is considered the “fingerprint” of biomass [22]. This peak, which is very sharp in the raw material, weakens significantly and changes shape as the temperature increases (especially after 200 °C). This confirms that the hydrolysis reactions have successfully broken the cellulose and hemicellulose polymer chains. In the solid samples obtained, the presence of peaks around 1600–1620 cm^−1^ (C=C aromatic ring vibrations) and 1700 cm^−1^ (C=O carbonyl groups) is preserved and becomes more pronounced at certain temperatures [23]. This finding indicates that during hydrothermal carbonization (HTC), biomass is not only decomposed but also transformed into a more stable carbon skeleton through aromatization and polymerization reactions.

### 3.4. Effect of Temperature and Pressure on 5-HMF and Furfural Yields

**Figure 7:**
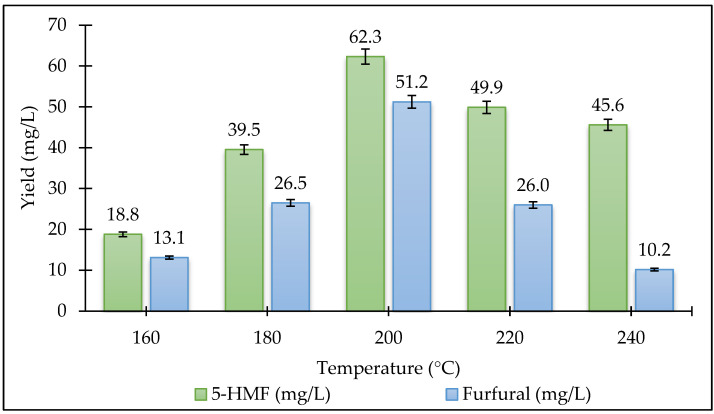
Variation of 5-HMF and furfural amount (mg/L) at different temperatures (0 MPa, 15 min).

**Figure 8:**
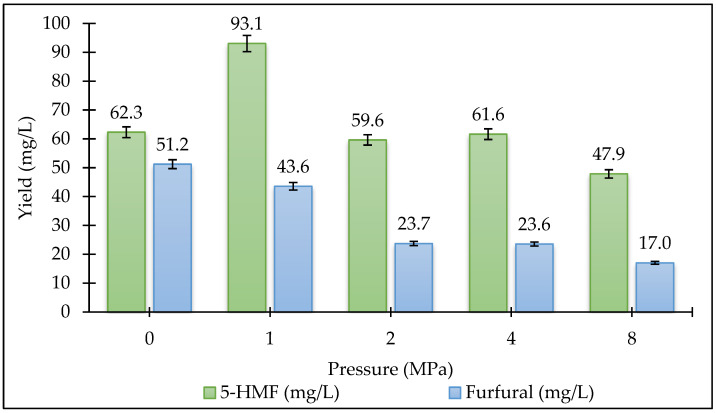
Variation of 5-HMF and furfural amount (mg/L) at different pressures (200 °C, 15 min).

In this study, product formation was expressed in terms of liquid phase concentrations (mg/L) instead of mass-based recovery yields. Since the reaction volume (150 mL) and initial biomass loading (10 g, on a dry basis) were kept constant throughout all experiments, the concentration values provide a consistent and directly comparable metric for evaluating the effects of temperature and pressure. The primary objective of this study is to investigate the relative effect of process parameters and establish statistically significant trends, rather than to determine absolute mass recovery efficiencies. Therefore, concentration-based data were considered suitable for comparative statistical modeling under constant experimental conditions. The reaction temperature and ambient pressure are effective parameters in all thermochemical conversion processes. The effects of temperature and pressure on the amount of 5-HMF and furfural in the HTL reaction are shown in Figure 7 and Figure 8. The study used five different temperatures: 160 °C, 180 °C, 200 °C, 220 °C, and 240 °C. Experiments were conducted at four different pressures of 1, 2, 4, and 8 MPa at the temperature where the 5-HMF and furfural amount was optimum. Our results indicated that the conversion of *Stevia rebaudiana* to 5-HMF and furfural via the HTL reaction is significantly influenced by temperature. When the reaction temperature was increased from 160 °C to 200 °C, the yield increased significantly, but it decreased at higher temperatures. The highest yields of 5-HMF (93.1 mg/L) and furfural (51.2 mg/L) were achieved at 200 °C, beyond which degradation pathways dominated, likely due to rehydration to levulinic acid and humin formation. The decline in 5-HMF yield above 200 °C aligns with prior reports on its thermal instability, where rehydration or condensation reactions dominate [24]. The sharp decline in 5-HMF yield above 200 °C aligns with studies on carbohydrate-rich biomass, where excessive heat promotes acid-catalyzed side reactions [25]. To mitigate this, future work could employ water-tolerant Lewis acids [26] or biphasic solvents [27] to stabilize 5-HMF. Biphasic solvent systems (e.g., water/THF) may enhance 5-HMF recovery by minimizing side reactions [27]. Additionally, biphasic systems can also enhance the recovery of furfural by reducing vapor phase losses; this has been validated as a strategy for other volatile platform chemicals. When examining the effect of pressure on the 5-HMF amount, the highest value was obtained at 1 MPa. When the pressure increased, the yield decreased significantly. However, the furfural efficiency has decreased as the pressure increased. The highest furfural efficiency was achieved when the initial pressure was zero. Considering all these results, the highest 5-HMF amount (93.1 mg/L) was obtained at a temperature of 200 °C and a pressure of 1 MPa. The highest amount of furfural (51.2 mg/L) was obtained at a temperature of 200 °C and a pressure of 0 MPa. The lowest 5-HMF amount (18.8 mg/L) was obtained at a temperature of 160 °C with an initial pressure of zero. The lowest furfural amount (10.2 mg/L) was obtained at a temperature of 240 °C and a pressure of 0 MPa. While 5-HMF yields peaked at 1 MPa (93.1 mg/L), furfural production was maximized at ambient pressure (51.2 mg/L), reflecting its volatility and susceptibility to condensation under pressurized conditions [28].

### 3.5. ANOVA and Model Coefficient Evaluation for 5-HMF Yield

The results of the General Factorial Regression analysis performed to determine the effect of temperature and pressure parameters on 5-HMF production yield are presented in Table 3. The established regression model was found to be statistically highly significant at a 95% confidence interval (Fmodel = 11.38, *p* = 0.001). This indicates that the selected parameters successfully explain the variation in the experimental output.

When examining the ANOVA results, it is observed that the temperature factor has a very high F-value (F = 24.41, *p* < 0.05). This finding proves that, between the two studied variables, reaction temperature is the more dominant and decisive parameter on the 5-HMF yield. In contrast, the F-value calculated for the pressure parameter is 1.34, and the *p*-value is 0.32 (*p* > 0.05). This result indicates that pressure variation within the studied range of 0–4 MPa does not create a statistically significant difference in 5-HMF formation.

When the model coefficients are detailed, it is observed that the coefficient for the 160 °C temperature level is negative (−22.23, *p* = 0.000), while the coefficient for the 200 °C temperature level is positive and reaches the highest value (+25.98, *p* = 0.000). This confirms that increasing the temperature up to 200 °C positively affects yield. Furthermore, the fact that the variance inflation factor (VIF) values are at a low level of 1.5 for all terms indicates that there is no multicollinearity problem in the model and that the coefficient estimates are reliable.

**Figure 9:**
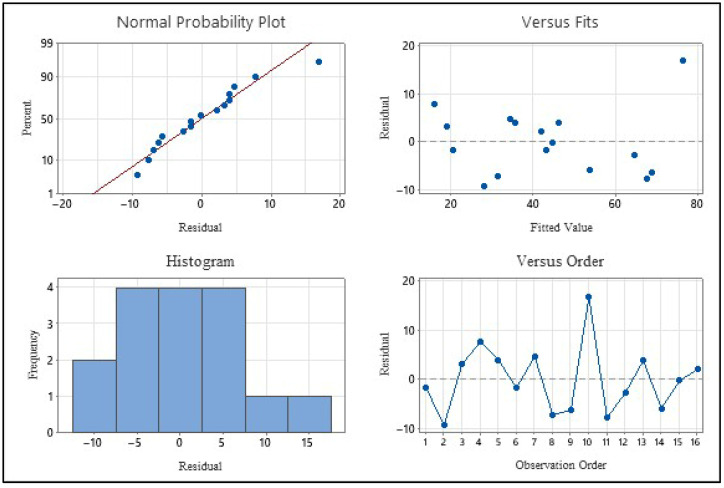
Residual Plots for 5-HMF (mg/L).

The predictive power of the model and its fit to experimental data were evaluated using the determination coefficients (R^2^) and standard error (S) values. According to the analysis results, the model’s R^2^ value was calculated as 88.35%. This value indicates that 88.35% of the total variation in 5-HMF yield can be explained by the temperature and pressure parameters (and their interactions). The adjusted coefficient of determination (R^2^adj) was found to be 80.59%, indicating that the model has been stripped of unnecessary variables. The predictive coefficient of determination (R^2^pred), which indicates the model’s ability to predict the results of new experiments, was determined to be 63.19%. Considering the complex structure of experimental biomass conversion processes, this value demonstrates that the model has acceptable predictive capability. Furthermore, the standard error (S) value, calculated as 8.75, indicates that the deviation of the experimental data from the regression curve is within reasonable limits. The suitability of the developed statistical model to the ANOVA hypothesis was examined through residual analysis graphs and is presented in Figure 9.

The normal probability graph shown in Figure 9 indicates that the residuals are almost perfectly linearly arranged on the reference line. Additionally, the fact that the histogram graph generally has a bell curve (Gaussian distribution) shape confirms that the error terms meet the assumption of normal distribution. When examining the residual versus fit graph, it can be seen that the data points are randomly and irregularly distributed around the zero axis. The absence of any funnel or distinct pattern supports the assumption that the model’s variance is constant. In the versus order graph, it was observed that the data fluctuated randomly without showing a systematic increase or decrease trend. This proves that any systematic error arising from the order in which the experiments were conducted or any external factor (correlation) that changed over time did not affect the results. The analyses have shown that the established model is statistically robust and that the obtained ANOVA results can be confidently used to predict experimental data.

### 3.6. ANOVA and Model Coefficient Evaluation for Furfural Yield

The statistical analysis results (ANOVA) of the effects of temperature and pressure on furfural yield are presented in Table 4. The established general factorial regression model was found to be statistically significant at a 95% confidence interval (Fmodel = 7.44, *p* = 0.004). This result indicates that the model successfully explains the experimental variation in the furfural production process.

When process parameters are examined, temperature is seen to be the main factor determining furfural yield (F = 14.51, *p* = 0.001). When analyzing the temperature coefficients, it was determined that the 160 °C level has a negative effect (−13.01) on yield, while the 200 °C level has a positive and the highest effect (+12.42). This confirms that increasing temperature promotes furfural formation up to a certain point. In contrast, the *p*-value for the pressure parameter was calculated as 0.775 (*p* > 0.05), indicating that, similar to the 5-HMF results, pressure variation had no statistically significant effect on furfural yield.

**Figure 10:**
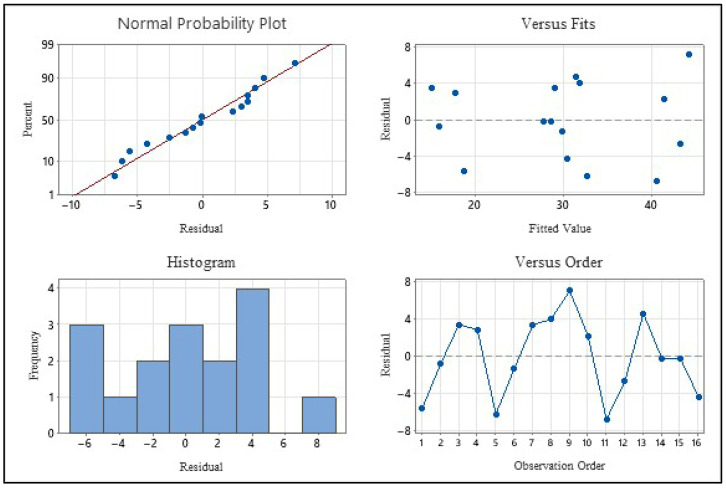
Residual Plots for Furfural (mg/L).

When evaluating the model’s performance indicators, the coefficient of determination (R^2^) was found to be 83.22%. However, the fact that the predicted coefficient of determination (R^2^pred) remained at 46.96% can be attributed to furfural having a more complex reaction mechanism compared to 5-HMF. Furfural’s tendency to polymerize at high temperatures and transform into humic structures, as well as the unpredictability of secondary degradation reactions, partially limit the model’s predictive capacity. Nevertheless, the obtained R^2^ value indicates that the model is sufficient to explain the general trend of the experimental data. The suitability of the regression model created for furfural yield to the ANOVA assumptions has been verified through residual analysis graphs (Figure 10).

When examining the normal probability plot, it can be seen that the residuals are arranged with nearly ideal linearity, without deviation from the red reference line. The distribution in the histogram graph also generally exhibits a symmetrical structure, indicating that the errors successfully meet the normality assumption. In the residuals versus fits graph, it is observed that the data points are randomly distributed along the horizontal axis and do not form any expanding megaphone or distinct curvilinear pattern. This situation indicates that the model’s variance is constant (homoscedasticity) and that the error size is independent of the estimated value. The residuals versus order graph shows that the data fluctuates randomly according to the order of the experiments. The absence of any systematic increase or decrease trend proves that there was no device-related deviation or external factor (time series effect) during the experiments.

Although the model’s prediction coefficient (R^2^ pred) is lower compared to the 5-HMF model, the residual analysis confirms that the model is statistically robust and that the discussed parameter effects are reliable.

## 4. Conclusions

In addition to furan formation, the hydrothermal process has led to the simultaneous production of biochar and bio-oil fractions. Although the primary focus of this study is the evaluation of 5-HMF and furfural formation under varying temperature and pressure conditions, the formation of these by-products highlights the multi-product nature of the hydrothermal conversion pathway. The presence of solid and liquid fractions indicates the potential for integrated biomass valorization within a biorefinery framework. The hydrothermal processing of lignocellulosic biomass was carried out at temperatures of 160 °C, 180 °C, 200 °C, 220 °C, and 240 °C, and pressures of 1, 2, 4, and 8 MPa. The primary objective was the production of 5-HMF and furfural from the *Stevia rebaudiana* plant through hydrothermal liquefaction. A significant effect of temperature and pressure on the quantities of 5-HMF and furfural was observed. Additionally, an HPLC method was developed to determine these compounds. This method exhibits excellent resolution, wide linear ranges, and favorable LOD and LOQ values. Compared to existing HPLC methods for 5-HMF/furfural analysis, our approach offers three key advantages: (1) a shorter runtime (40 min vs. 60 min in conventional methods), (2) higher sensitivity and (3) compatibility with complex biomass matrices without extensive sample cleanup. Recent studies have also highlighted the need for rapid, robust HPLC methods in biorefinery applications [24,29], and our protocol addresses these demands effectively. In conclusion, the validated HPLC method presented here provides a reliable, high-throughput solution for quantifying 5-HMF and furfural in *Stevia rebaudiana*-derived bio-oils. Future work could explore coupling this method with mass spectrometry (LC-MS) for enhanced sensitivity or testing alternative columns for improved separation of polar byproducts.

This study demonstrates a hydrothermal conversion approach for the utilization of the *Stevia rebaudiana* plant. Given the increasing demand for natural sweeteners, the extraction volume of *Stevia rebaudiana* leaves is expected to rise. Furthermore, the development of high-value products from the residual biomass after plant refinement could represent a significant advancement. The study shows that *Stevia rebaudiana* waste can be utilized to obtain valuable compounds. Statistical analyses have shown that, between the two parameters studied (temperature and pressure), temperature has a stronger effect on the yields of both 5-HMF and furfural. The formation of 5-HMF and furfural increased with temperature up to 200 °C, while further increases to 220 °C and 240 °C led to a decrease in yields, likely due to increased thermal decomposition and the formation of secondary byproducts. This behavior confirmed that the process could best be described using quadratic models. In conclusion, it has been demonstrated that the conversion of the *Stevia rebaudiana* plant into 5-HMF and furfural compounds is feasible under hydrothermal conditions. In future studies, the variation in 5-HMF and furfural quantities could be investigated by altering reaction parameters and incorporating catalysts.

## Figures and Tables

**Table 1:** Recovery, regression equation, standard deviation, limit of detection and quantification for 5-HMF.

Standard Concentration (mg/L)	Measured Concentration (mg/L)	Recovery (%)
2.5	2.39	95.47
5	4.84	96.81
10	10.76	107.57
25	24.61	98.42
50	50.78	101.57
100	102.95	102.95
250	262.65	105.06
500	499.34	99.87
	Accuracy	100.96 ± 4.14
	Linearity Range (mg/L)	2.5–500
	Correlation Coefficient	0.9997
	SE of Intercept	2.0378
	SD of Intercept	5.7637
	LOD (mg/L)	6.6841
	LOQ (mg/L)	20.2550

**Table 2:** Recovery, regression equation, standard deviation, limit of detection and quantification for furfural.

Standard Concentration (mg/L)	Measured Concentration (mg/L)	Recovery (%)
2.5	2.53	101.12
5	4.97	99.37
10	11.37	113.69
25	26.88	107.54
50	54.65	109.30
100	104.82	104.82
250	250.50	100.20
500	498.45	99.69
	Accuracy	104.47 ± 5.29
	Linearity Range (mg/L)	2.5–500
	Correlation Coefficient	0.9997
	SE of Intercept	0.9519
	SD of Intercept	2.6924
	LOD (mg/L)	3.1597
	LOQ (mg/L)	9.5747

**Table 3:** Statistical analysis (ANOVA) of the effects of temperature and pressure parameters on 5-HMF yield.

**Analysis of Variance**					
**Source**	**DF**	**Adj SS**	**Adj MS**	**F-Value**	***p*** **-Value**
**Model**	6	5233.4	872.24	11.38	0.001
**Linear**	6	5233.4	872.24	11.38	0.001
**Temperature**	3	4924.1	1641.38	21.41	0
**Pressure**	3	309.3	103.1	1.34	0.32
**Error**	9	690	76.66		
**Total**	15	5923.4			
**Coefficients**					
**Term**	**Coef**	**SE Coef**	**T-Value**	***p*** **-Value**	**VIF**
**Constant**	43.19	2.19	19.73	0	
**Temperature (°C)**					
**160**	−22.23	3.79	−5.86	0	1.5
**180**	−7.12	3.79	−1.88	0.093	1.5
**200**	25.98	3.79	6.85	0	1.5
**Pressure (MPa)**					
**0**	−0.55	3.79	−0.14	0.888	1.5
**1**	7.13	3.79	1.88	0.093	1.5
**2**	−1.8	3.79	−0.48	0.646	1.5

DF: Degrees of Freedom, Adj SS: Adjusted Sum of Squares, Adj MS: Adjusted Mean Square, F-Value: Fisher’s variance ratio, *p*-Value: Probability value, Coef: Coefficient, SE Coef: Standard Error of the Coefficient, T-Value: Student’s t-statistic, VIF: Variance Inflation Factor. The highest levels of the factors.

**Table 4:** Statistical analysis (ANOVA) of the effects of temperature and pressure parameters on Furfural yield.

**Analysis of Variance**					
**Source**	**DF**	**Adj SS**	**Adj MS**	**F-Value**	***p*** **-Value**
**Model**	6	1331.98	222	7.44	0.004
**Linear**	6	1331.98	222	7.44	0.004
**Temperature**	3	1298.66	432.89	14.51	0.001
**Pressure**	3	33.31	11.1	0.37	0.775
**Error**	9	268.59	29.84		
**Total**	15	1600.57			
**Coefficients**					
**Term**	**Coef**	**SE Coef**	**T-Value**	***p*** **-Value**	**VIF**
**Constant**	29.86	1.37	21.86	0	
**Temperature (°C)**					
**160**	−13.01	2.37	−5.5	0	1.5
**180**	0.95	2.37	0.4	0.696	1.5
**200**	12.42	2.37	5.25	0.001	1.5
**Pressure (MPa)**					
**0**	1.85	2.37	0.78	0.454	1.5
**1**	−0.96	2.37	−0.4	0.696	1.5
**2**	−1.79	2.37	−0.76	0.469	1.5

DF: Degrees of Freedom, Adj SS: Adjusted Sum of Squares, Adj MS: Adjusted Mean Square, F-Value: Fisher’s variance ratio, *p*-Value: Probability value, Coef: Coefficient, SE Coef: Standard Error of the Coefficient, T-Value: Student’s t-statistic, VIF: Variance Inflation Factor. The highest levels of the factors.

## Data Availability

The original contributions presented in this study are included in the article. Further inquiries can be directed to the corresponding author.
